# Novel cut‐off values of time from diagnosis to systematic therapy predict the overall survival and the efficacy of targeted therapy in renal cell carcinoma: A long‐term, follow‐up, retrospective study

**DOI:** 10.1111/iju.14751

**Published:** 2021-11-30

**Authors:** Chuanzhen Cao, Jianzhong Shou, Hongzhe Shi, Weixing Jiang, Xiangpeng Kang, Ruiyang Xie, Bingqing Shang, Xingang Bi, Jin Zhang, Shan Zheng, Aiping Zhou, Changling Li, Jianhui Ma

**Affiliations:** ^1^ Department of Urology National Cancer Center/National Clinical Research Center for Cancer/Cancer Hospital Chinese Academy of Medical Sciences and Peking Union Medical College Beijing China; ^2^ Department of Radiology National Cancer Center/National Clinical Research Center for Cancer/Cancer Hospital Chinese Academy of Medical Sciences and Peking Union Medical College Beijing China; ^3^ Department of Pathology National Cancer Center/National Clinical Research Center for Cancer/Cancer Hospital Chinese Academy of Medical Sciences and Peking Union Medical College Beijing China; ^4^ Department of Medical Oncology National Cancer Center/National Clinical Research Center for Cancer/Cancer Hospital Chinese Academy of Medical Sciences and Peking Union Medical College Beijing China

**Keywords:** metastasectomy, metastatic renal cell carcinoma, prognostic factor, survival, targeted therapy

## Abstract

**Objectives:**

Metastatic renal cell carcinoma can occur synchronously or metachronously. We characterized the time from diagnosis to systematic therapy as a categorical variable to analyze its effect on the overall survival and first‐line treatment efficacy of metastatic renal cell carcinoma patients.

**Methods:**

We initially enrolled 949 consecutive metastatic renal cell carcinoma patients treated with targeted therapies retrospectively from December 2005 to December 2019. X‐tile analysis was used to determine cut‐off values of time from diagnosis to systematic therapy referring to overall survival. Patients were divided into different groups based on the time from diagnosis to systematic therapy and then analyzed for survival.

**Results:**

Of 358 eligible patients with metastatic renal cell carcinoma, 125 (34.9%) had synchronous metastases followed by cytoreductive nephrectomy, and 233 (65.1%) had metachronous metastases. A total of 28 patients received complete metastasectomy. Three optimal cut‐off values for the time from diagnosis to systematic therapy (months) – 1.1, 7.0 and 35.9 – were applied to divide the population into four groups: the synchro group (time from diagnosis to systematic therapy ≤1.0), early group (1.0 < time from diagnosis to systematic therapy ≤ 7.0), intermediate group (7.0 < time from diagnosis to systematic therapy < 36.0) and late group (time from diagnosis to systematic therapy ≥36.0). The targeted therapy‐related overall survival (*P* < 0.001) and progression‐free survival (*P* < 0.001) values were significantly different among the four groups. Patients with longer time from diagnosis to systematic therapy had better prognoses and promising efficacy of targeted therapy. With the prolongation of time from diagnosis to systematic therapy, complete metastasectomy was more likely to achieve and bring a better prognosis.

**Conclusions:**

The time from diagnosis to systematic therapy impacts the survival of metastatic renal cell carcinoma patients treated with targeted therapy. The cutoff points of 1, 7 and 36 months were statistically significant. The statistical boundaries might be valuable in future model establishment.

**Abbreviations & Acronyms**
BMI: body mass index
CFDA: China Food and Drug Administration
CI: confidence interval
ICI: immune checkpoint inhibitor
IMDC: International Metastatic Renal Cell Carcinoma Database Consortium
KPS: Karnofsky Performance Status
mRCC: metastatic renal cell carcinoma
MSKCC: Memorial Sloan‐Kettering Cancer Center
NCC/CHCAMS: National Cancer Center/Cancer Hospital, Chinese Academy of Medical Sciences
NCCN: National Comprehensive Cancer Network
OS: overall survival
PDGFR: platelet‐derived growth factor receptor
PFS: progression‐free survival
RCC: renal cell carcinoma
TDT: time from diagnosis to systematic therapy
TKI: tyrosine kinase inhibitor
tOS: therapy‐related overall survival

## Introduction

RCC accounts for approximately 3% of all malignant tumors, and its incidence is increasing worldwide.^1^, ^2^ Approximately 35% of RCC patients present with mRCC or relapse after radical or partial nephrectom.y.^3^ Untreated patients with mRCC have a poor prognosis, with a 5‐year OS rate of <10%.^4^ With the improved understanding of related molecular mechanisms, especially the role of angiogenesis, the treatment of mRCC has progressed dramatically in recent times. Treatments that target angiogenesis have shown significant improvements in the prognosis of mRCC.

Although more urologists and oncologists have focused on the efficacy of immune therapy or immune therapy combined with targeted therapy for mRCC since ICIs were recently discovered to yield large improvements in disease outcomes, targeted therapy remains the cornerstone of systematic treatment for mRCC. According to the current guidelines of the NCCN (version 1.2020), the first‐line therapies for mRCC include sunitinib, pazopanib, cabozantinib, ICIs or ICIs combined with axitinib.^5^ Although ICIs have been shown to improve the survival of mRCC patients,^6^ sunitinib (Sutent; Pfızer, New York, NY, USA) and pazopanib (Votrient; Glaxo, Brentford, UK) are the first‐line regimens approved by the CFDA, and the data on long‐term prognosis are mainly based on targeted therapies. Sunitinib is an orally administered TKI targeting several receptors, including vascular endothelial growth factor receptor types 1–3, PDGFR‐α, PDGFR‐β, c‐KIT and FMS‐like tyrosine kinase.^7^ Pazopanib is another tyrosine kinase inhibitor targeting the same receptors, except for FMS‐like tyrosine kinase.^8^ Both tyrosine kinase inhibitors improve the prognosis of mRCC in terms of OS or PFS, according to the results of randomized phase III trials.^8^, ^9^, ^10^, ^11^ Despite promising survival benefits, the prognosis of mRCC remains poor. Therefore, the stratification of patients who would benefit from first‐line therapy is important. Since the era of targeted therapy, many prognostic factors and predictors of treatment efficacy have been analyzed. The IMDC risk stratification model was constructed based on the MSKCC risk stratification guidelines,^12^ and has been widely adopted for mRCC patients treated with targeted therapies.^13^ Among the prognostic factors of both models, KPS and laboratory variables (hemoglobin, calcium, neutrophil, platelet or lactate dehydrogenase) focus on the patient’s physical condition, whereas TDT is the unique factor reflecting tumor biological characteristics.

Therefore, it is important to explore the value of TDT in prognosis prediction. However, the cut‐off values for TDT vary among different studies. IMDC‐related research showed that patients had a worse prognosis if the TDT was <1 year.^13^ In contrast, cut‐offs of 2 years or 5 years have also showed different oncologic outcomes.^14^, ^15^, ^16^ All the time boundaries were selected arbitrarily with no certificated data to support the cut‐off value.^12^, ^13^, ^14^, ^15^, ^16^ Therefore, we thought it would be valuable to analyze the relationship between TDT and OS in mRCC patients. In the present study, we investigated the clinicopathological features of RCC patients with metastases at any time after nephrectomy, and then made TDT a categorical variable to determine the ability of TDT to predict the OS and first‐line treatment efficacy of mRCC patients.

## Methods

The present study was approved by the Ethics Committee of the NCC/CHCAMS (ID: 20/245‐2441). Patient consent for treatment and follow up was included in each medical record. We aimed to evaluate the predictive value of TDT on the prognosis and efficacy of first‐line targeted therapy in RCC patients.

**Fig. 1:**
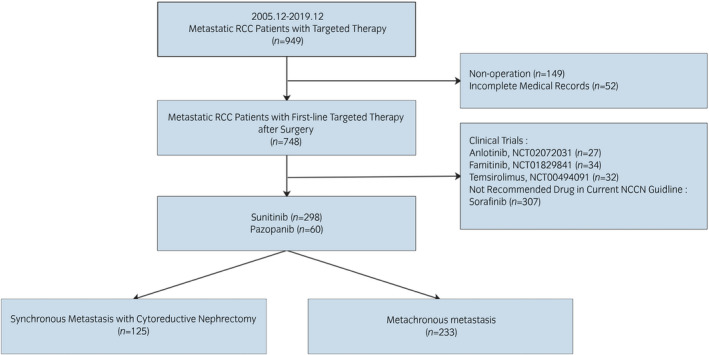
Process of screening patients.

We initially enrolled 949 consecutive mRCC patients treated with targeted therapies in NCC/CHCAMS, from December 2005 to December 2019. A total of 358 patients received nephrectomy for RCC, suffered metastasis synchronously or metachronously and received first‐line targeted therapy.^5^ Others with incomplete medical records or enrolled clinical trials were excluded. As previous studies showed different primary tumor volumes influenced the survival of mRCC patients, we excluded synchronous mRCC patients who had not received cytoreductive nephrectomy to avoid the influence of primary tumor load on outcomes.^17^, ^18^, ^19^ The process of patient screening is shown in Figure 1.

The demographic and clinicopathological data of patients, such as age, sex, BMI, laterality, symptoms and signs, tumor size, T and N staging, pathology type, sarcomatoid differentiation, tumor necrosis, renal vein tumor thrombus, Fuhrman grade, and laboratory results, were obtained from the Electronic Medical Record System. The KPS score and IMDC risk stratification were evaluated at the time metastasis was detected. Surgery was carried out using standard methods for open or laparoscopic nephrectomy. Retroperitoneal lymphadenectomy was carried out when regional lymph node enlargement was observed by imaging or during surgery. All pathologies were reviewed according to the 2016 World Health Organization urinary system and male genital organ histological classification criteria.^20^ The pathological stage was determined by the American Joint Committee on Cancer tumor, node, metastasis staging system (8th edition). The sites of metastasis were evaluated by imaging examinations, such as computed tomography, nuclear magnetic resonance, ultrasound, radionuclide bone imaging or positron emission tomography. All patients received sunitinib or pazopanib as first‐line targeted therapy. Sunitinib was administered at a dosage of 50 mg daily on a schedule of 4 weeks on‐2 weeks off, and pazopanib was administered at a dosage of 800 mg once daily. Treatment was continued until disease progression or severe toxicity occurred. Standard second‐line treatments or clinical trials were applied after sunitinib or pazopanib treatment failed.

Follow‐up evaluation consisted of physical examination, laboratory tests and imaging examination. Survival information was obtained through outpatient record review and telephone follow ups. All follow ups were concluded on 1 November 2020.

### Statistical analysis

The date of initial diagnosis was defined as the date of histological confirmation by nephrectomy (partial, radical or cytoreductive). TDT was defined as the time from diagnosis to targeted therapy. OS was defined as the time from diagnosis to the date of death from any cause or the last follow up. Targeted tOS was defined as the time from targeted therapy to the date of death from any cause or the last follow up. PFS was defined as the time from the onset of targeted therapy to the date of disease progression or last follow up. Kaplan–Meier analysis was used to analyze survival rates, as well as the median survival time. Univariable and multivariable COX proportional hazards regression models were used to determine prognostic and independent factors. Factors significant in univariable analysis were evaluated using multivariable models. X‐tile 3.6.1 software (Yale University, New Haven, CT, USA) was used to determine the optimal cut‐off values for dividing the population into four groups (synchro, early, intermediate and late), and OS was used as the end‐point.^21^ The log‐rank test was used to estimate differences between the survival curves. A non‐parametric test was used to compare variables of groups. Statistical analysis was carried out using spss statistics (version 23.0; IBM Corporation, Armonk, NY, USA), and differences were considered statistically significant if *P* < 0.05.

## Results

A total of 358 eligible patients were identified. The median age was 56 years, and 79.9% were men (M : F = 286:72). A total of 68 patients (19.0%) had clinical symptoms, including hematuria (*n* = 44, 64.7%), backache (*n* = 18, 26.5%) or fever (*n* = 6, 8.8%). Among them, 312 (87.1%) were diagnosed with clear cell type, 19 (5.3%) were diagnosed with papillary type, four (1.1%) were diagnosed with chromophobe type, 22 (6.1%) were diagnosed with unclassified type and one (0.3%) was diagnosed with collecting duct carcinoma. Sunitinib was administered to 298 patients (83.2%), and pazopanib was administered to 60 patients (16.8%). A total of 28 patients received complete metastasectomy. After two or three cycles of first‐line targeted therapy, the metastases of 28 patients were considered operable evaluated by imaging, and metastasectomy was carried out followed by targeted therapy. The frequent sites of metastasis were the lung (*n* = 264, 73.7%), bone (*n* = 90, 25.1%), liver (*n* = 24, 6.7%), adrenal gland (*n* = 24, 6.7%) and brain (*n* = 11, 3.1%). There were 224 (62.6%) and 134 (37.4%) patients who had metastases with single organ and multiple organs, respectively. There were 116 (32.4%) patients in the favorable‐risk group, 138 (38.5%) in the intermediate‐risk group and 104 (29.1%) in the poor‐risk group according to IMDC risk stratification. Clinical–pathological characteristics are summarized in Table 1.

**Table 1:** Patient characteristics and correlation between the TDT and clinicopathological features of patients with RCC

Clinicopathological factors	Total	TDT (months)				χ^2^	*P*‐value
		≤1.0	1.0–7.0	7.0–36.0	≥36.0		
No. patients (*n*, %)	358	128, 35.8%	51, 14.2%	92, 25.7%	87, 24.3%	–	–
Age, years (median)	56	59	56	54	52	23.23	<0.001
Sex (*n*)							
Male	286	108	41	67	70	4.47	0.215
Female	72	20	10	25	17		
BMI, kg/m^2^ (median)	24.1	24.3	24.1	24.2	24.1	4.21	0.240
Laterality (*n*)							
Left	179	72	32	36	39	10.56	0.141
Right	179	56	19	56	48		
Clinical symptoms (*n*)							
Yes	68	24	19	17	8	16.46	<0.001
No	290	104	32	75	79		
Maximum tumor size, cm (median)	4.5	4.5	6.0	4.5	4.9	18.58	<0.001
Blood transfusion (*n*)							
Yes	45	24	9	8	4	10.55	0.014
No	313	104	42	84	83		
Pathology type (*n*)							
Clear cell	312	103	43	80	86	10.69	0.014
Non‐clear cell	46	25	8	12	1		
T staging (*n*)							
1	143	30	18	39	56	38.09	<0.001
2	29	17	3	4	6		
3	156	56	29	48	23		
4	30	25	1	1	3		
N staging (*n*)							
0	305	90	45	85	85	37.31	<0.001
1	53	38	6	7	2		
Fuhrman grade (*n*)							
1	8	0	1	1	6	54.82	<0.001
2	113	25	10	34	44		
3	181	66	29	51	35		
4	56	37	11	6	2		
Sarcomatoid differentiation (*n*)							
Yes	38	21	6	10	1	12.78	0.005
No	320	107	45	82	86		
Tumor necrosis (*n*)							
Yes	163	72	34	32	25	29.21	<0.001
No	195	56	17	60	62		
Renal vein tumor thrombus (*n*)							
Yes	54	24	7	14	9	2.94	0.402
No	304	104	44	78	78		
Complete metastasectomy (*n*)							
Yes	28	1	2	10	15	21.71	<0.001
No	330	127	49	82	72		
Percentage of complete metastasectomy	7.82%	0.78%	3.92%	10.87%	17.24%		
Hemoglobin, g/L (median)	133	131	128	125	140	13.84	0.003
Neutrophil, ×10^9^/L (median)	4.0	4.0	4.5	3.5	3.5	14.10	0.003
Platelet, ×10^9^/L (median)	201.0	201.0	107.0	198.0	201.0	6.34	0.096
Calcium, mmol/L (median)	2.30	2.28	2.24	2.29	2.30	6.17	0.103
KPS at metastasis, score (mean)	82.3	78.2	80.4	84.0	85.8	71.38	<0.001
Organ number of metastasis (*n*)							
Single	224	88	30	53	53	3.45	0.327
Multiple	134	40	21	39	34		
IMDC risk group (*n*)							
Favorable	116	0	3	47	66	172.27	<0.001
Intermediate	138	52	34	36	16		
Poor	104	76	14	9	5		
Targeted drug (*n*)							
Sunitinib	298	114	41	74	69	4.87	0.181
Pazopanib	60	14	10	18	18		

All patients were followed up successfully with a median follow‐up time of 102.6 months (range 4.1–321.1 months). The median OS was 52.2 months (95% CI 44.1–60.2 months), with a 5‐year OS of 45.2% and a 10‐year OS of 31.2%. The median PFS was 14.8 months (95% CI 12.9–16.7 months). The median tOS was 32.9 months (95% CI 27.9–37.8 months). The TDT ranged from 0 to 274.8 months. A total of 18 factors were significant in univariable COX regression analysis (*P* < 0.05). We carried out multivariable COX analysis for these 18 factors. BMI (*P* < 0.001), non‐clear cell RCC (*P* = 0.031), T staging (*P* = 0.001), tumor necrosis (*P* = 0.009), thrombocytosis (*P* = 0.001), KPS (*P* = 0.010), IMDC (*P* < 0.001 and *P* = 0.001) and TDT (*P* < 0.001) were independent prognostic factors identified (Table 2).

**Table 2:** Univariate and multivariable Cox proportional hazards regression analyses of OS

Factors	Univariate			Multivariate		
	Hazard ratio	95% CI	*P*‐value	Hazard ratio	95% CI	*P*‐value
Age (>56 years)	1.61	1.23–2.11	0.001			
Sex (male)	0.98	0.71–1.36	0.918			
BMI (>23.9 km/m^2^)	0.49	0.38–0.65	<0.001	0.56	0.42–0.75	<0.001
Laterality (left)	1.26	0.97–1.65	0.083			
Clinical symptoms (yes)	1.88	1.38–2.56	<0.001			
Maximum tumor size (>7 cm)	1.26	0.93–1.71	0.132			
Blood transfusion (yes)	2.01	1.42–2.84	<0.001			
Non‐clear cell RCC (yes)	2.40	1.65–3.48	<0.001	1.52	1.04–2.23	0.031
T staging (T3/4)	2.47	1.86–3.27	<0.001	1.61	1.21–2.16	0.001
N staging (N1)	3.24	2.33–4.49	<0.001			
Fuhrman grade (3/4)	2.65	1.94–3.62	<0.001			
Sarcomatoid differentiation (yes)	2.79	1.91–4.08	<0.001			
Tumor necrosis (yes)	2.07	1.58–2.70	<0.001	1.50	1.10–2.03	0.009
Renal vein tumor thrombus (yes)	1.65	1.17–2.31	0.004			
Organ number of metastasis (multiple)	0.97	0.96–1.72	0.851			
Anemia (yes)	2.22	1.68–2.94	<0.001			
Neutrophil (>7.5 × 10^9^/L)	0.73	0.59–0.90	0.003			
Platelet (>300 × 10^9^/L)	2.51	1.84–3.43	<0.001	2.03	1.34–3.09	0.001
Calcium (>2.5 mmol/L)	2.64	1.86–3.75	<0.001			
KPS at metastasis (score <80)	2.86	2.09–3.90	<0.001	1.62	1.12–2.33	0.010
IMDC risk group						
Favorable	Reference			Reference		
Intermediate	5.08	3.40–7.60	<0.001	2.32	1.46–3.68	<0.001
Poor	11.14	7.38–16.81	<0.001	2.73	1.51–4.93	0.001
TDT	0.95	0.94–0.96	<0.001	0.96	0.96–0.97	<0.001

As OS was used as the end‐point, X‐tile defined three optimal cut‐off values for TDT – 1.1, 7.0 and 35.9 months – and converted the continuous variable to a categorical variable. The analysis results of X‐tile are shown in Figure S1.

Four related groups, the synchro group (TDT ≤1.0, 128 patients), early group (1.0 < TDT ≤ 7.0, 51 patients), intermediate group (7.0 < TDT < 36.0, 92 patients) and late group (TDT ≥36.0, 87 patients), were constructed. The clinicopathological characteristics of the four groups are summarized in Table 1. The variables of age (*P* < 0.001), clinical symptoms (*P* < 0.001), maximum tumor size (*P* < 0.001), blood transfusion (*P* = 0.014), pathology type (*P* = 0.014), T staging (*P* < 0.001), N staging (*P* < 0.001), Fuhrman grade (*P* < 0.001), sarcomatoid differentiation (*P* = 0.005), tumor necrosis (*P* < 0.001), complete metastasectomy (*P* < 0.001), KPS at metastasis (*P* < 0.001) and IMDC risk group (*P* < 0.001) were all significantly different among the four groups.

**Fig. 2:**
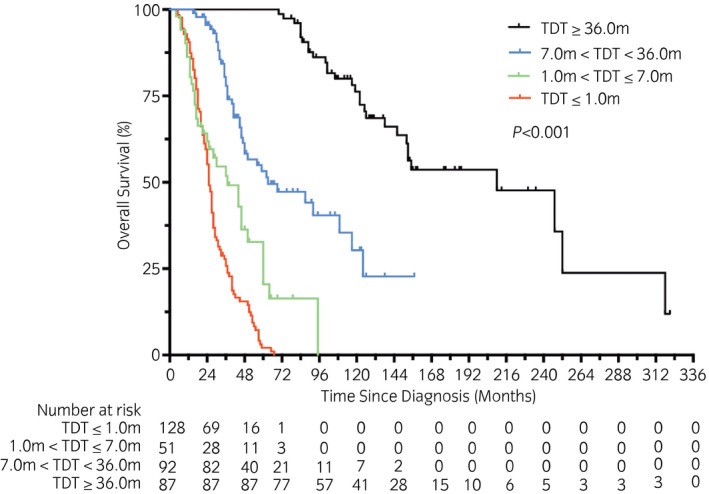
Kaplan–Meier curves with log‐rank statistics for OS in patients of four different groups.

After population stratification, the median OS times of the four groups were 25.0 months (synchro, 95% CI 22.8–27.2), 37.0 months (early, 95% CI 19.8–54.1), 62.9 months (intermediate, 95% CI 27.3–98.4) and 209.9 months (late, 95% CI 133.4–286.5), respectively. The 5‐year OS rates were 2.1% (synchro), 20.5% (early), 53.2% (intermediate) and 100.0% (late), respectively (*P* < 0.001, χ^2^ = 256.3; Fig. 2). In addition, according to IMDC risk, an additional log‐rank test was carried out. The median OS times were 152.8 months (favorable risk, 95% CI 79.0–226.5), 40.2 months (intermediate risk, 95% CI 32.2–48.1) and 26.0 months (poor risk, 95% CI 22.6–29.4), respectively (*P* < 0.001, χ^2^ = 169.7; Fig. S2).

**Fig. 3:**
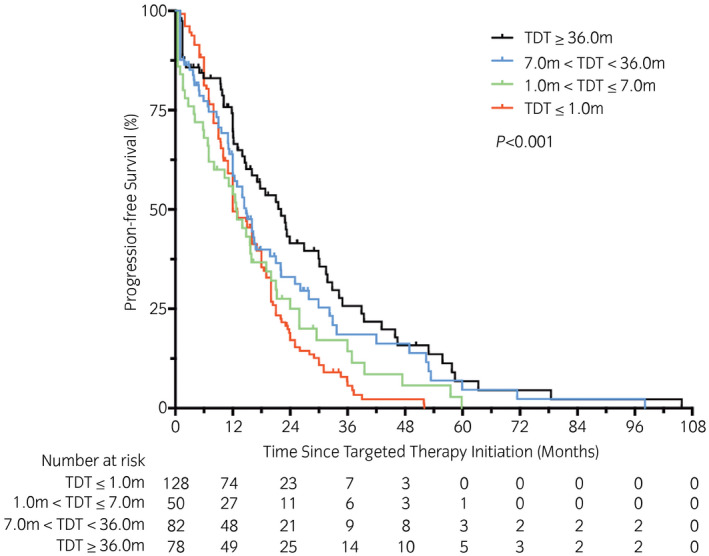
Kaplan–Meier curves with log‐rank statistics for PFS in patients of four different groups.

All patients were administered first‐line targeted therapy after confirmation of metastasis. As 20 patients had not undergone regular imaging follow ups because of self‐factors, the actual time of disease progression could not be evaluated. Therefore, 338 cases were enrolled to carry out PFS analysis. The median PFS times stratified according to TDT groups were 12.0 months (synchro, 95% CI 9.6–14.4), 12.7 months (early, 95% CI 8.7–16.7), 14.8 months (intermediate, 95% CI 11.7–17.9) and 22.1 months (late, 95% CI 15.7–28.5), respectively. The 3‐year PFS rates were 5.6% (synchro), 14.3% (early), 18.6% (intermediate) and 25.7% (late). The 5‐year PFS rates were 0.0% (synchro and early), 4.6% (intermediate) and 6.8% (late), respectively (*P* < 0.001, χ^2^ = 16.56; Fig. 3).

**Fig. 4:**
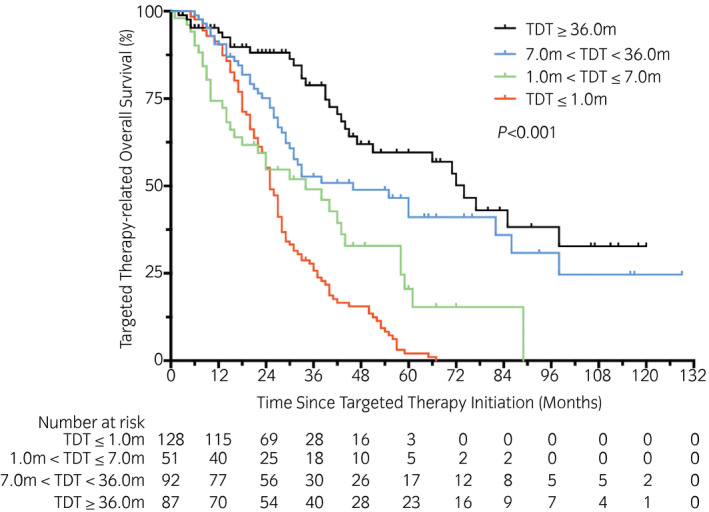
Kaplan–Meier curves with log‐rank statistics for targeted tOS in patients of four different groups.

The median tOS times were 25.0 months (synchro, 95% CI 22.8–27.2), 33.9 months (early, 95% CI 17.5–50.4), 46.1 months (intermediate, 95% CI 19.2–73.0) and 74.4 months (late, 95% CI 62.2–86.7), and the 5‐year tOS rates were 2.1%, 20.5%, 41.1% and 59.6%, respectively. The tOS curves of the four groups were also significantly different according to the log‐rank test (*P* < 0.001, χ^2^ = 73.98; Fig. 4). Up to the last follow up, 220 patients died because of cancer progression. The results of log‐rank tests in each two group regarding OS, PFS and tOS are shown in Table S1.

**Fig. 5:**
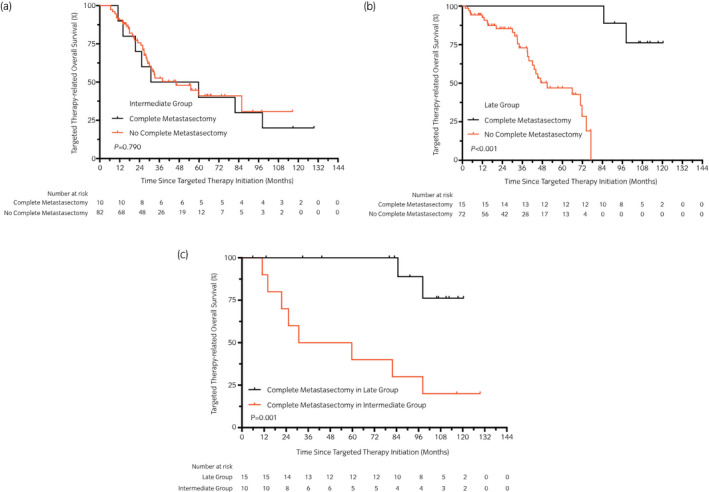
Targeted tOS of patients with complete metastasectomy or not in different groups. (a) Log‐rank statistics of patients in the intermediate group comparing complete metastasectomy with no complete metastasectomy. (b) Log‐rank statistics of patients in the late group comparing complete metastasectomy with no complete metastasectomy. (c) Targeted tOS curves of patients with complete metastasectomy in the intermediate and late groups.

During data analyses, an intriguing finding showed that more patients were likely to achieve complete metastasectomy as TDT became longer. Among the 28 patients who underwent complete metastasectomy, there was one in the synchro group, two in the early group, 10 in the intermediate group and 15 in the late group (*P* < 0.001). Further survival analyses were carried out. There was no difference in tOS between these patients in the intermediate group (*P* = 0.790; Fig. 5a), but patients in the late group had better tOS if complete metastasectomy was carried out (median tOS: NA *vs* 50.9 months; *P* < 0.001, χ^2^ = 18.06; Fig. 5b). Patients with complete metastasectomy in the late group had a better tOS than those in the intermediate group (median tOS: NA *vs* 45.3 months; *P* = 0.001, χ^2^ = 10.21; Fig. 5c). It seemed patients with longer TDT had a better prognosis if a complete metastasectomy was carried out.

## Discussion

RCC metastasis can appear at any time, simultaneously or heterochronously from the time of diagnosis. Although targeted therapies have benefited mRCC patients in recent times, a predictive marker to predict the prognoses of patients receiving targeted therapies is required. In the present study, we converted the TDT from a continuous variable to a categorical variable based on OS, resulting in four groups divided by three cut‐off values: 1.1, 7.0 and 35.9 months. As the TDT became longer, the corresponding patients had longer OS. This result shows that TDT is a significant predictor of OS. TDT can also predict the efficacy of mRCC first‐line targeted therapy in PFS and tOS.

The prognostic predictive effect of TDT has been assessed in several studies, but the boundaries vary in different studies. The MSKCC and IMDC risk score models are validated prognostic models and have become increasingly important for patient selection for systemic treatments. The IMDC risk model adopts the concept of the TDT referring to the MSKCC model.^13^ However, the boundary of 1 year in the IMDC risk model might not be appropriate. The median value of the TDT (range 0–31.6 months) was calculated as 1.4 years. Finally, 1 year was used instead of 1.4 years to divide the population into two groups, and the study showed that a time from diagnosis to the initiation of therapy of <1 year was an independent predictor of short survival (median OS: 15.8 months *vs* 30.9 months, *P* = 0.0098).^13^ Retrospective research carried out by the Cleveland Clinic showed another prognostic model adopting a TDT cut‐off of 2 years.^16^ With a median follow‐up time of 10.9 months for 120 patients, patients who experienced recurrence within 2 years had worse PFS than those who experienced recurrence beyond 2 years (9.2 months *vs* 19.9 months, *P* = 0.009). A longer TDT boundary of 5 years was used in another retrospective study.^15^ All the time boundaries were selected with no certificated statistical data to support the cut‐off value.^12^, ^13^, ^14^, ^15^, ^16^ The biggest difference from previous studies is that we used statistical analysis (X‐tile) to explore the intervals of TDT. Thus, the survival results might be more objective with this patient stratification.

Although different studies used different time boundaries, TDT is indeed a significant independent predictor for mRCC patients. In our research, BMI (*P* < 0.001), non‐clear cell RCC (*P* = 0.031), T staging (*P* = 0.001), tumor necrosis (*P* = 0.009), thrombocytosis (*P* = 0.001), KPS (*P* = 0.010), IMDC (*P* < 0.001 and *P* = 0.001) and TDT (*P* < 0.001) were independent prognostic factors. These factors had been verified as independent factors of mRCC in many studies previously, except for TDT.^22^, ^23^, ^24^ Related studies tended to artificially transfer TDT to a categorical variable for analysis, such as 1 year, 2 years and so on.^13^, ^14^, ^15^, ^16^ However, we confirmed the TDT, a continuous variable, was an independent factor by multivariable Cox analysis and divided patients into four groups according to the cut‐off values of TDT. Furthermore, a longer TDT predicted better survival and efficacy of targeted therapies. Furthermore, patients with longer TDT also had more favorable related variables, such as clinical symptoms, maximum tumor size, T staging, N staging, Fuhrman grade, sarcomatoid differentiation, tumor necrosis, KPS at metastasis and IMDC risk.

Currently, RCC patients with synchronous metastases are defined as those with detected metastases at the initial visit, and these patients have poor prognoses. As the CARMENA trial indicated, cytoreductive nephrectomy followed by sunitinib did not improve the prognosis of mRCC and resulted in a poor median OS of 13.9 months.^25^ The SURTIME trial explored whether sunitinib therapy before cytoreductive nephrectomy improved outcome compared with immediate surgery followed by sunitinib. The results showed immediate surgery did not benefit outcome, which was consistent with the CARMENA study. However, the SURTIME trial showed that with the deferred cytoreductive nephrectomy, more patients received sunitinib and OS results were higher than the intermediate group (*P* = 0.03).^26^ A retrospective study analyzed the OS and PFS benefits of cytoreductive nephrectomy in synchronous mRCC patients treated with targeted therapies. In total, 982 synchronous mRCC patients received cytoreductive nephrectomy. With a median follow‐up time of 39.1 months, mRCC patients with cytoreductive nephrectomy had a better median OS (20.6 months *vs* 9.5 months, *P* < 0.001) and median PFS (7.6 months *vs* 4.5 months, *P* < 0.001) than those without cytoreductive nephrectomy.^27^ Another similar study enrolled 294 synchronous mRCC patients, 189 of whom received cytoreductive nephrectomy. This result showed that cytoreductive nephrectomy improved OS in synchronous mRCC patients treated with targeted therapies, and the median OS was 23.0 months.^28^ In the present study, 125 patients received cytoreductive nephrectomy before first‐line targeted therapy. As grouped by X‐tile analysis, one cut‐off value of TDT was defined as 1.1 months rather than 0 (referring to OS), and three RCC patients with metachronous metastasis within 1 month were classified as the synchro group together with the synchronous mRCC patients. The median tOS was 25.0 months, which was slightly better than that in previous studies, but still poorer than that in other groups. According to the present data, we considered that it might be feasible to modify the definition of synchronous metastasis as metastasis occurring simultaneously or within 1 month.

Several studies and meta‐analyses are available to evaluate the evidence for metastasectomy in the treatment of mRCC.^29^, ^30^, ^31^, ^32^ Recently, Lyon *et al*. analyzed the value of complete metastasectomy for mRCC patients in the era of targeted therapy. Of 586 patients diagnosed with mRCC, 158 were treated with complete metastasectomy. With a median follow‐up time of 3.9 years, the investigators found that complete metastasectomy was associated with a reduced likelihood of death from RCC (*P* < 0.001).^32^ In our subgroup analyses, a similar but more detailed finding was that complete metastasectomy had a different prognostic value in the intermediate and late groups. In the late group, patients who received complete metastasectomy had better tOS than those who did not receive complete metastasectomy (*P* = 0.001). However, this difference was not found in the intermediate group (*P* = 0.790). The subgroup analysis showed the complete metastasectomy might be more likely to be achieved in metachronous mRCC patients with longer TDT and perhaps benefit prognosis. However, just 7.8% (28/358) of patients experienced complete resection of metastasis, and the results of subgroup analyses require verification by further research.

The limitations to the present study include its retrospective design. Another limitation was that the population focused on first‐line targeted therapy with sunitinib or pazopanib. In addition, the sample consisted of a heterogeneous population in which just 46 patients had non‐clear cell RCC, and just 28 patients received complete metastasectomy.

The TDT impacts the survival of mRCC patients treated with targeted therapy. The boundaries of 1, 7 and 36 months were statistically significant. The statistical boundaries might be valuable in future model establishment. This might need to be taken into consideration in further studies, and could help in patient stratification and prognostication.

## Author contributions

Chuanzhen Cao: Conceptualization; Data curation; Formal analysis; Methodology; Project administration; Resources; Software; Validation; Writing – original draft; Writing – review & editing. Jianzhong Shou: Conceptualization; Funding acquisition; Project administration; Resources; Supervision. Hongzhe Shi: Conceptualization; Project administration; Resources; Supervision; Visualization; Writing – review & editing. Weixing Jiang: Methodology; Resources; Software; Validation; Visualization. Xiangpeng Kang: Data curation; Methodology; Resources; Software. Ruiyang Xie: Data curation; Formal analysis; Methodology; Software. Bingqing Shang: Data curation; Methodology; Resources. Xingang Bi: Data curation; Validation; Visualization. Jin Zhang: Data curation; Investigation; Software. Shan Zheng: Data curation; Formal analysis; Resources. Aiping Zhou: Supervision; Validation; Visualization. Changling Li: Conceptualization; Investigation; Project administration. Jianhui Ma: Investigation; Project administration; Supervision.

## Conflict of interest

None declared.

## Approval of the research protocol by an Institutional Reviewer Board

This study was approved by the Ethics Committee of the National Cancer Center/Cancer Hospital, Chinese Academy of Medical Sciences (ID: 20/245‐2441).

## Informed consent

Patient informed consent for treatment and follow up was included in each medical record.

## Registry and the Registration No. of the study

N/A.

## Animal studies

N/A.

## Supporting information


**Figure S1.** X‐tile analysis was carried out in 358 patients referring to OS. The continuous variable, TDT (months), was calculated, resulting in three optimal cut‐off values as 1.1, 7.0 and 35.9. The patients were divided into four groups according to the cut‐off values, and the related Kaplan–Meier curve had statistical difference (*P* < 0.001).

**Figure S2.** Kaplan–Meier curves with log‐rank statistics for OS in patients of different IMDC risk groups.

**Table S1.** Log‐rank tests of OS, tOS and PFS among subgroup.
