# Supplementary figures and images for: Novel cut‐off values of time from diagnosis to systematic therapy predict the overall survival and the efficacy of targeted therapy in renal cell carcinoma: A long‐term, follow‐up, retrospective study
Source: Int J Urol. 2021 Nov 30;29(3):212–20. doi: 10.1111/iju.14751 (PMC9299735; doi:10.1111/iju.14751)

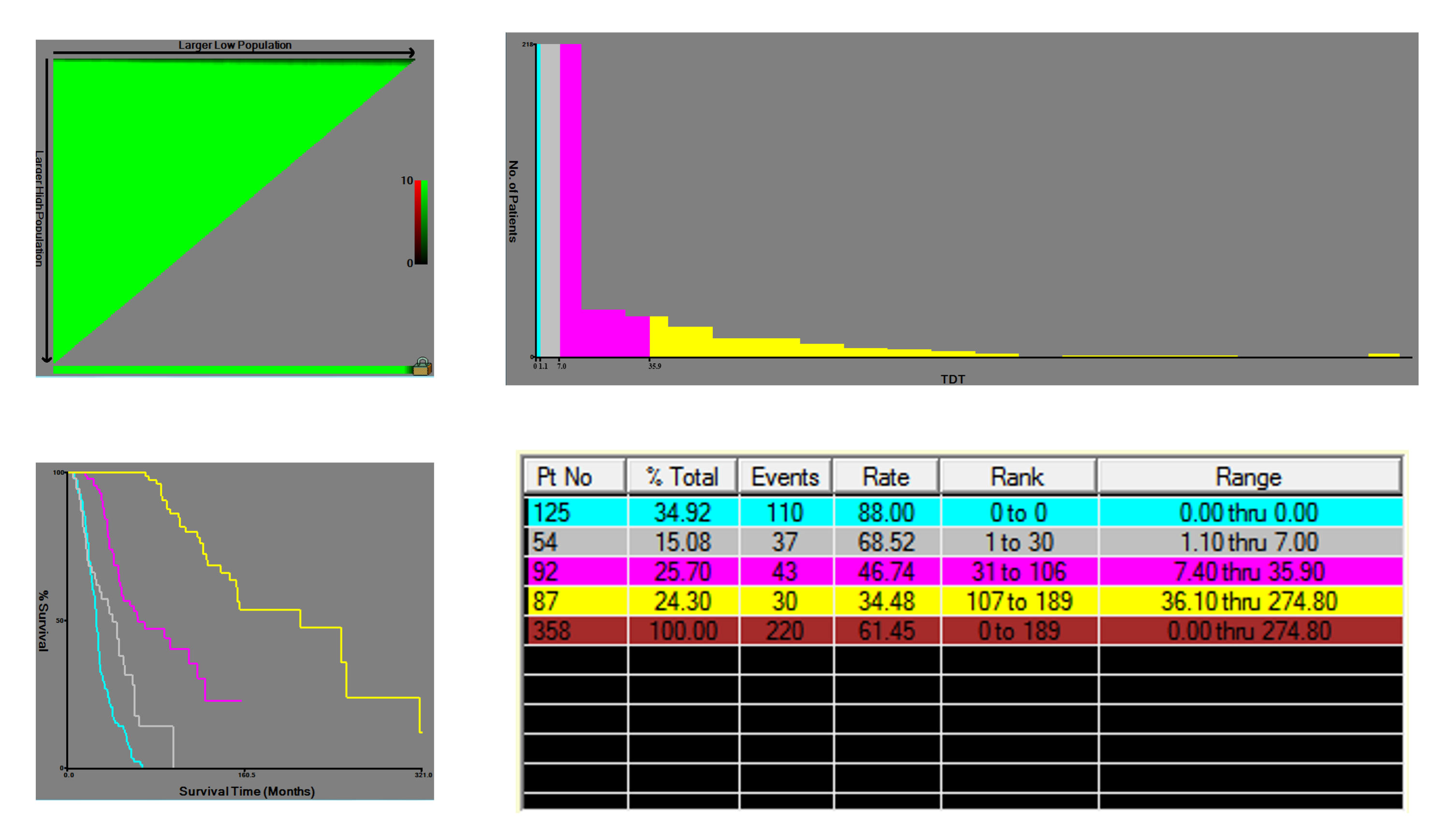

Supplement: Supplementary file 1 — Figure S1. X‐tile analysis was carried out in 358 patients referring to OS. The continuous variable, TDT (months), was calculated, resulting in three optimal cut‐off values as 1.1, 7.0 and 35.9. The patients were divided into four groups according to the cut‐off values, and the related Kaplan–Meier curve had statistical difference (P < 0.001). [file IJU-29-212-s003.tiff]

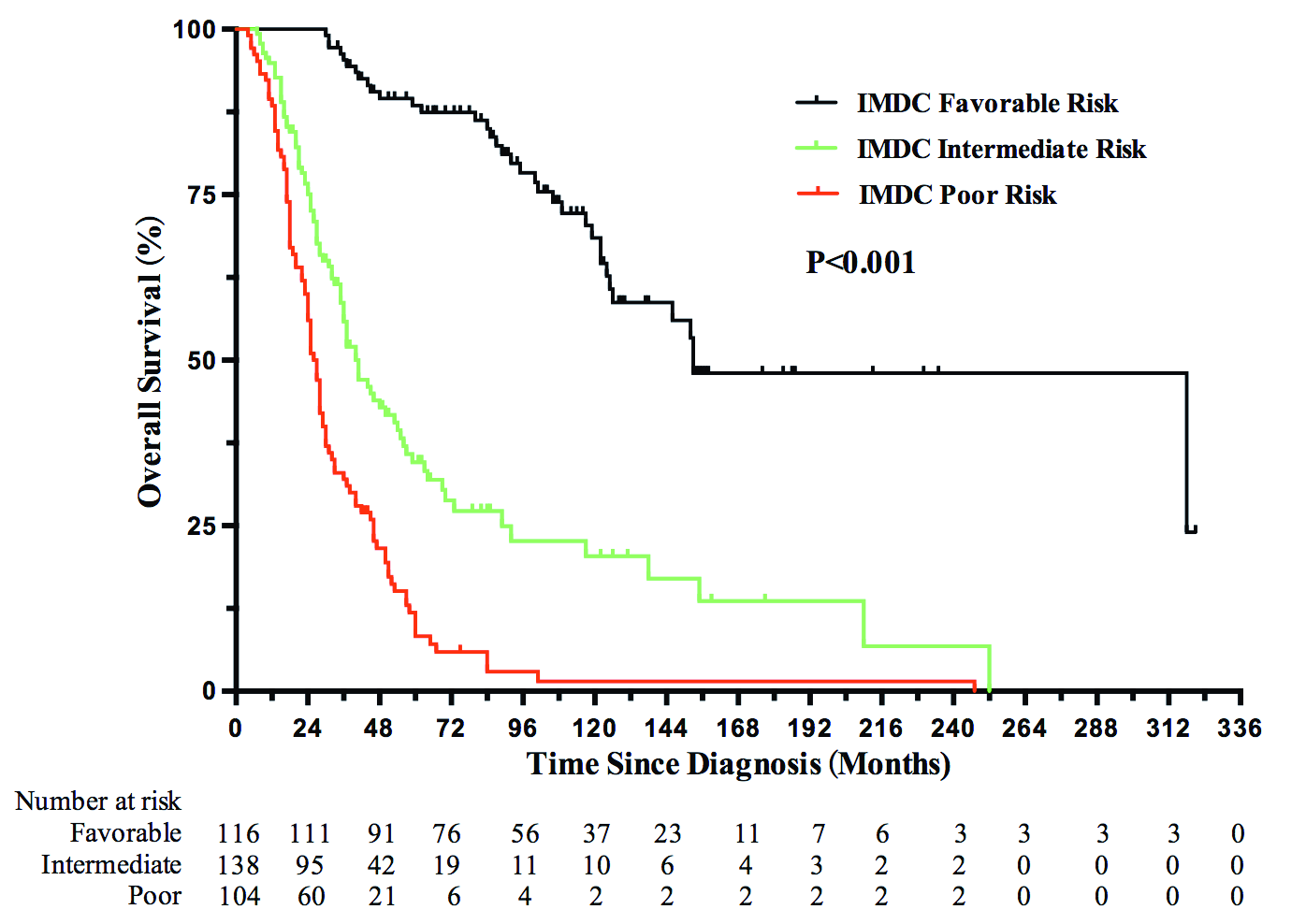

Supplement: Supplementary file 2 — Figure S2. Kaplan–Meier curves with log‐rank statistics for OS in patients of different IMDC risk groups. [file IJU-29-212-s002.tiff]
