# Supplementary material for: Novel cut‐off values of time from diagnosis to systematic therapy predict the overall survival and the efficacy of targeted therapy in renal cell carcinoma: A long‐term, follow‐up, retrospective study
Source: Int J Urol. 2021 Nov 30;29(3):212–20. doi: 10.1111/iju.14751 (PMC9299735; doi:10.1111/iju.14751)
Supplement: Supplementary file 3 — Table S1. Log‐rank tests of OS, tOS and PFS among subgroup. [file IJU-29-212-s001.docx]

Table s1. Log-rank tests of OS, tOS and PFS among subgroups

|  |  | Median (month) | χ^2^ | P Value |
| --- | --- | --- | --- | --- |
| OS |  |  |  |  |
|  | Synchro vs Early | 25 vs 37 | 10.84 | 0.001 |
|  | Early vs Intermediate | 37 vs 63 | 20.02 | <0.001 |
|  | Intermediate vs Late | 63 vs 210 | 46.51 | <0.001 |
| tOS |  |  |  |  |
|  | Synchro vs Early | 25 vs 34 | 6.35 | 0.012 |
|  | Early vs Intermediate | 34 vs 46 | 6.65 | 0.010 |
|  | Intermediate vs Late | 46 vs 74 | 3.96 | 0.047 |
| PFS |  |  |  |  |
|  | Synchro vs Early | 12 vs 13 | 0.40 | 0.526 |
|  | Early vs Intermediate | 13 vs 15 | 2.09 | 0.149 |
|  | Intermediate vs Late | 15 vs 22 | 2.05 | 0.152 |
